# Virtual Healthcare for Healthy Aging: Communication Shifts & Provider Satisfaction in the Pandemic

**DOI:** 10.1093/geroni/igaf122.846

**Published:** 2025-12-31

**Authors:** Su-I Hou

**Affiliations:** University of Central Florida, Orlando, Florida, United States

## Abstract

**Purpose:**

The COVID-19 pandemic significantly altered healthcare delivery, impacting provider interactions with older patients. This study examines shifts in communication methods and their effects on provider satisfaction.

**Methods:**

A survey including of 59 healthcare providers working with older patients in healthcare and long-term care settings analyzed communication modes before and during the pandemic. Open-ended questions explored provider experiences and recommendations for improving virtual interactions.

**Results:**

Before the pandemic, most communication occurred through in-office visits (41%), phone calls (10%), or a combination (32%), with high provider satisfaction (52% very satisfied, 39% satisfied). During the pandemic, over 70% of providers increased telemedicine and virtual communication, while in-office visits declined (27%), phone use increased (15%), email was introduced (5%), and mixed methods rose (44%). Provider satisfaction dropped significantly, with fewer than 25% very satisfied and 58% satisfied. Challenges included communication difficulties due to masks and face shields, as well as the need for better video systems and patient portal enrollment. While statistical significance was limited, qualitative findings emphasized provider skills and strong provider-patient relationships as key to effective care.

**Discussion:**

These findings highlight the need for further research with larger samples to assess the impact of virtual communication on provider satisfaction. The decline in satisfaction underscores the importance of training and support to optimize virtual healthcare interactions and ensure high-quality care for older clients.

